# The Application of the Design of Experiments and Artificial Neural Networks in the Development of a Fast and Straightforward HPLC-UV Method for Fluconazole Determination in Hemato-Oncologic Pediatric Patients and Its Adaptation to Therapeutic Drug Monitoring

**DOI:** 10.3390/ph17121679

**Published:** 2024-12-12

**Authors:** Arkadiusz Adamiszak, Andrzej Czyrski, Bartosz Sznek, Edmund Grześkowiak, Agnieszka Bienert

**Affiliations:** 1 Department of Clinical Pharmacy and Biopharmacy, Poznan University of Medical Sciences, 60-806 Poznan, Poland; arkadiusz.adamiszak@student.ump.edu.pl (A.A.); grzesko@ump.edu.pl (E.G.); 2 Doctoral School, Poznan University of Medical Sciences, 60-812 Poznan, Poland; 3 Department of Physical Pharmacy and Pharmacokinetics, Poznan University of Medical Sciences, 60-806 Poznan, Poland; aczyrski@ump.edu.pl (A.C.); bartosz.sznek@gmail.com (B.S.)

**Keywords:** HPLC-UV, human plasma, pharmacokinetics, method optimization, Box–Behnken design, central composite design, machine learning

## Abstract

**Objectives:** This study aimed to develop an optimized and wide concentration range HPLC-UV method for fluconazole (FLU) determination and its adaptation for pharmacokinetics (PK) studies in the pediatric population. **Methods:** The following parameters of chromatographic separation were optimized: retention time, tailing factor, peak height, and the sample preconditioning parameter, such as recovery. The optimization process involved the use of a central composite design (CCD) and Box–Behnken design (BBD) in the design of experiments (DoE) approach and a multilayer perceptron (MLP) for artificial neural network (ANN) application. Statistical and PK analyses were performed using Statistica and PKanalix. **Results:** The acetonitrile (ACN) concentration revealed the most significant factor influencing the retention time, tailing factor, and peak height of FLU and the internal standard. For recovery, the extracting agent’s volume was the most significant factor. In most cases, the analysis conducted with the DoE and ANN indicated the same factors in a similar order regarding their impact on the analyzed variables. The optimization process allowed for achieving a wide range of determined concentrations (0.5–100 mg/L) and ~100% recovery. The developed method enabled PK analysis of 12 samples from three pediatric patients, proving its clinical usability. The estimated median clearance (CL) and volume of distribution (Vd) were 1.01 L/h and 18.64 L, respectively. **Conclusions:** DoE and ANNs are promising and useful tools in the optimization of sample preconditioning as well as the HPLC separation procedure. The investigated fluconazole determination method meets the European Medicines Agency (EMA) validation objectives and might be used in pediatric and adult PK studies.

## 1. Introduction

Fluconazole (FLU), or 2-(2,4-difluorophenyl)-1,3-bis(1,2,4-triazol-1-yl)propan-2-ol, is an antifungal drug of the triazole group. Its antifungal activity is based on the selective inhibition of lanosterol 14α-demethylase, an essential enzyme for ergosterol biosynthesis in fungal cells. As a result, a lack of ergosterol leads to homeostasis dysfunctions and growth inhibition [1,2,3]. Regarding pharmacotherapy in the pediatric population, FLU is registered to treat invasive and mucosal candidiasis, cryptococcal meningitis, and prophylaxis of *Candida* spp. infections in immunocompromised patients and cryptococcal meningitis and high-risk patients [3,4].

The therapeutic activity of the FLU depends on its concentration and the minimum inhibitory concentration (MIC) of the pathogen. The most accurate pharmacokinetic/pharmacodynamic (PK/PD) target is a ratio of the area under the unbound drug concentration-time curve (ƒAUC) to the MIC equaling ~100 [5,6]. Routine FLU therapeutic drug monitoring (TDM) is not required in most cases. However, it might be reasonable for pediatric patients, severe fungal infections, and populations exposed to PK changes due to severe illness or complicated pharmacotherapy, such as oncological populations [5,6,7].

Many expensive and complicated mass spectroscopy FLU detection methods have been developed to measure extremely low FLU concentrations in different species, matrices, or even cellular fungi [8,9,10,11,12,13,14]. However, a question arises: Is HPLC with UV detection precise and accurate enough to perform TDM-based adjustments in pharmacotherapy? To answer this question, our study aimed to develop a financially rational, simple, fast, sensitive, selective, and precise HPLC method with ultraviolet (UV) detection for determining FLU in human plasma and its implementation in TDM in the hemato-oncologic pediatric population.

## 2. Results

### 2.1. Mobile Phase Optimization

The following parameters were optimized: the retention time, tailing factor, and FLU and IS peak heights. The Box-Behnken design was applied in the analysis, which was suitable for optimizing the mobile phase [15]. The statistical analysis confirmed the suitability of the model. The R^2^ value exceeded 0.98, and the R^2^_adj_ exceeded 0.95 (Table 1). The results of the ANOVA analysis are listed in Table 2, Table 3 and Table 4. The F value in the ANOVA analysis refers to the importance of the factor for the investigated process, and the *p* value refers to its significance. The higher the F value, the greater the impact on the process under study. The directly determined influence of the given factors on the studied parameter is demonstrated by the presented polynomial equations. The results of the experiments described below are presented in the Supplementary Materials (Table S1).

The following polynomial equations describe the retention time:

T_R, FLU_ = 5.538 − 2.573 × ACN − 0.517 × ACN^2^ + 0.022 × phosphates − 0.133 × phosphates^2^ + 0.071 × pH + 0.069 × pH^2^ + 0.014 × ACN × phosphates − 0.139 × ACN × pH + 0.020 × phosphates × pH

T_R, IS_ = 11.502 − 5.064 × ACN − 0.768 × ACN^2^ + 0.060 × phosphates − 0.244 × phosphates^2^ + 0.167 × pH + 0.120 × pH^2^ + 0.0169 × ACN × phosphates − 0.149 × ACN × pH + 0.063 × phosphates × pH

**Figure 1:**
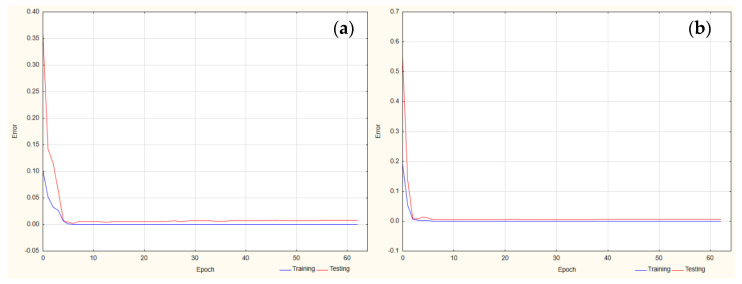
Learning curve for retention time analysis for (**a**) FLU and (**b**) IS.

According to the ANN approach, the best parameters were obtained for the retention time of FLU and the IS for the following MLP network (3-3-1). The subsequent activation functions were applied for FLU: logistic (in the hidden layer) and linear (in the output layer). For the IS, exponential and linear functions were used in the hidden and output layers, respectively (Figure 1).

The following polynomial equations describe the tailing factor:

T_f, FLU_ = 0.976 − 0.062 × ACN − 0.026 × ACN^2^ − 0.021 × phosphates + 0.016 × phosphates^2^ − 0.0452 × pH + 0.0002 × pH^2^ + 0.027 × ACN × phosphates − 0.025 × ACN × pH − 0.041 × phosphates × pH

T_f, IS_ = 1.056 − 0.022 × ACN − 0.008 × ACN^2^ − 0.010 × phosphates − 0.008 × phosphates^2^ − 0.002 × pH − 0.003 × pH^2^ − 0.001 × ACN × phosphates + 0.011 × ACN × pH − 0.017 × phosphates × pH

**Figure 2:**
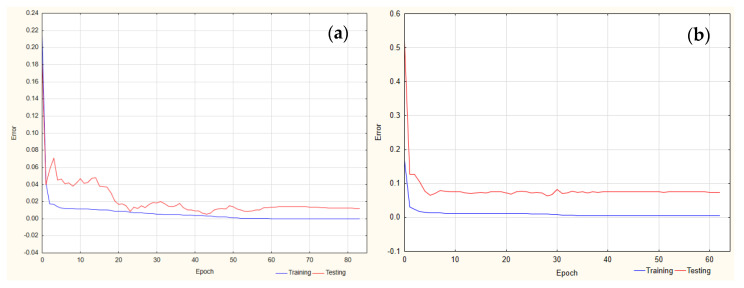
Learning curve for tailing factor analysis for (**a**) FLU and (**b**) IS.

In the case of a tailing factor of FLU and the IS, the best-trained ANNs were MLP (3-4-1) and MLP (3-5-1), respectively. The activation functions for analysis of the tailing FLU were hyperbolic tangent and linear functions (for the hidden and output layers, respectively). In the case of the IS, they were logistic and exponential (Figure 2).

The following polynomial equations describe the peak height:

Height, FLU = 21.280 + 8.024 × ACN − 0.450 × ACN^2^ − 0.352 × phosphates + 1.010 × phosphates^2^ − 1.988 × pH − 0.335 × pH^2^ + 0.925 × ACN × phosphates − 2.486 × ACN × pH − 0.610 × phosphates × pH

Height, IS = 15.503 + 5.565 × ACN − 0.395 × ACN^2^ − 0.246 × phosphates + 1.069 × phosphates^2^ − 2.009 × pH − 0.489 × pH^2^ + 0.698 × ACN × phosphates − 2.738 × ACN × pH − 0.590 × phosphates × pH

**Figure 3:**
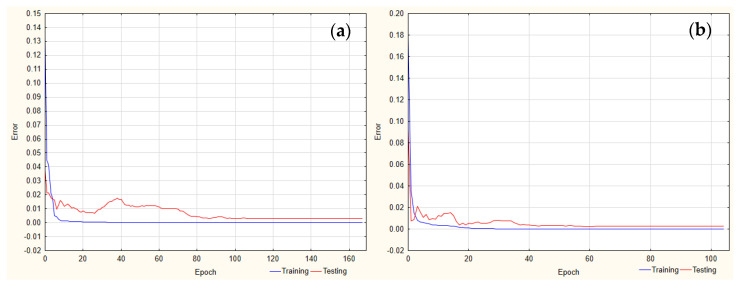
Learning curve for peak height analysis for (**a**) FLU and (**b**) IS.

In the case of the peak height, the best-trained ANN for FLU was observed for five neurons in the hidden layer (MLP 3-5-1), with the logistic function used as an activation function in both layers. For the IS, it was three neurons (MLP 3-3-1), with a logistic function in the hidden layer and a linear function in the output layer (Figure 3).

### 2.2. LLE Optimization

The statistical analysis confirmed the suitability of CCD—the R^2^ was 0.9977, and the R^2^_adj_ was 0.9947. The lack of fit was not significant. According to statistical analysis, the most significant factors influencing recovery were VDCM and the pH level. For these variables, the F values were the highest. The results of the statistical analysis are presented in Table 5. The results of the experiments are presented in the Supplementary Materials (Table S2).

The following polynomial equations describe the peak height:

Recovery_FLU_ = 86.555 + 7.064 × VDCM − 0.721 × VDCM^2^ − 3.204 × pH + 1.298 × pH^2^ − 2.345 × time + 3.895 × VDCM × time − 3.603 × pH × time

**Figure 4:**
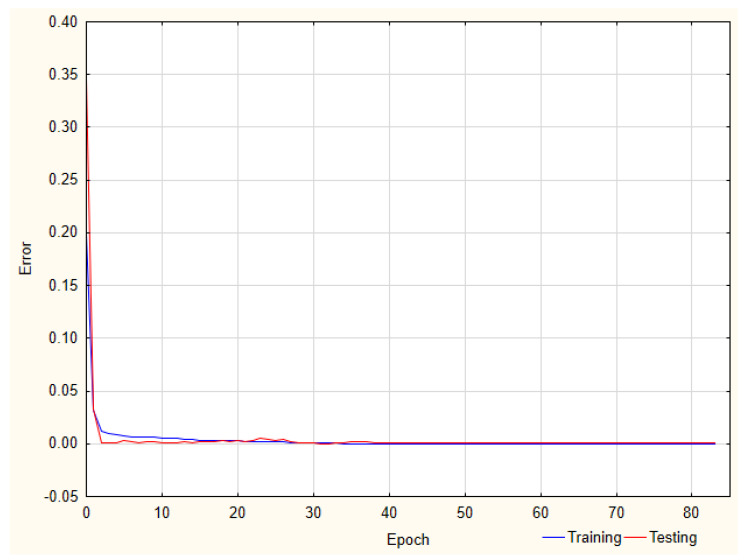
Learning curve for the FLU recovery analysis.

For the recovery of FLU, the best performance was observed for the ANN with four neurons in the hidden layer (MLP 3-4-1) and the following activation functions: hyperbolic tangent and linear in the hidden and output layer, respectively (Figure 4).

### 2.3. The Validation Parameters

The validation parameters proved that the method is suitable for bioanalysis. The method was linear within the analyzed concentration range, and the correlation coefficient exceeded 0.9995. The intra- and interday CV and RE were lower than 11% (Table 6). The FLU and IS met the stability criteria in each tested condition except for 1 year at −25 °C and −80 °C (Table 7). However, for the 1 year stability, the RE was still lower than 25%. The recovery reached 98.82% for 1.5 and 99.12% for 75.0 mg/L FLU concentrations.

### 2.4. Patient Sample Analysis and Pharmacokinetics

**Figure 5:**
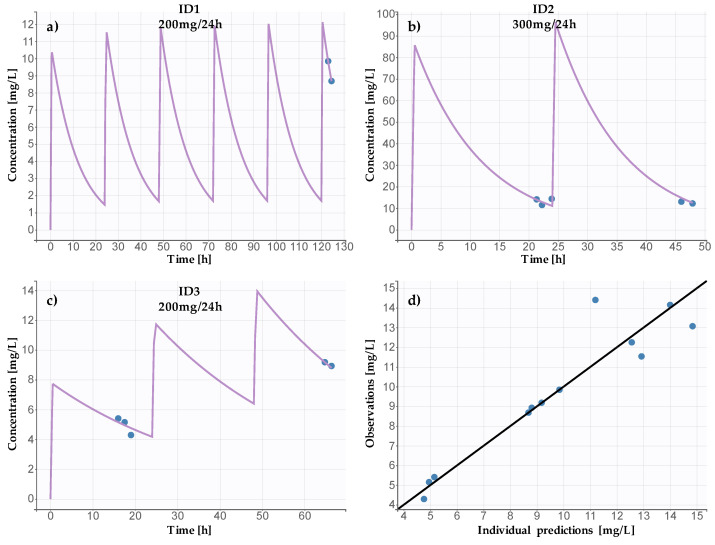
Patient PK profiles, showing (**a**) ID1, (**b**) ID 2, (**c**) ID3, and (**d**) observations vs. predicted concentrations plot. The purple lines represent individual PK profiles and the blue dots measured FLU concentrations.

According to the results of the PK analysis, the median of the estimated CL was equal to 1.01 L/h (IQR: 0.48–1.42), and the estimated Vd reached 18.64 L (IQR: 7.22–33.56), which is consistent with the results presented by Seay et al. and the PK information from SmPC [4,16]. The individual PK profiles of the investigated patients and the diagnostic plots are presented in Figure 5.

## 3. Discussion

### 3.1. Mobile Phase Optimization

#### 3.1.1. Retention Time

**Figure 6:**
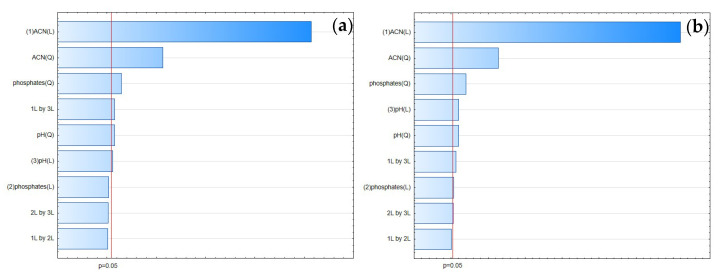
The Pareto chart for the retention times for (**a**) FLU and (**b**) the IS.

**Figure 7:**
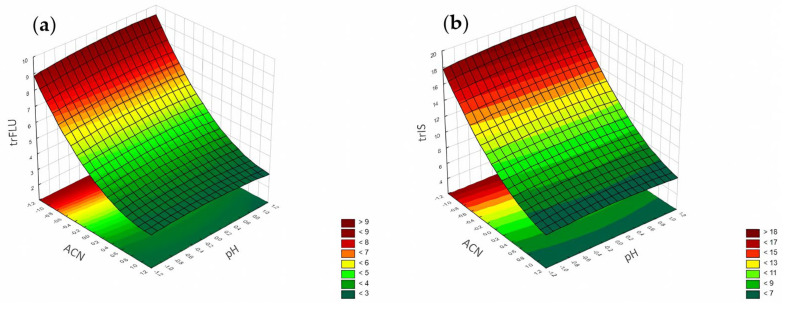
The RSM diagram for analysis of the retention time when analyzing ACN and the pH for (**a**) FLU and (**b**) the IS.

**Figure 8:**
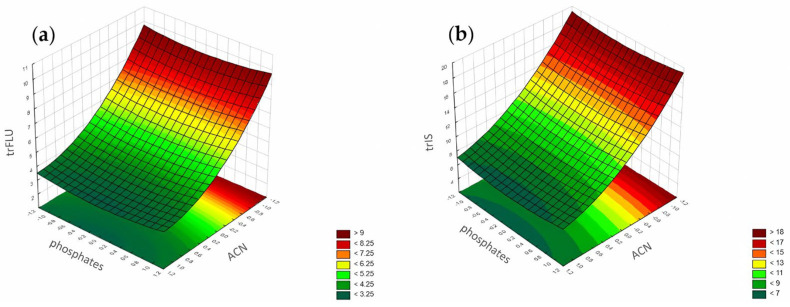
The RSM diagram for analysis of the retention time when analyzing ACN and phosphates for (**a**) FLU and (**b**) the IS.

**Figure 9:**
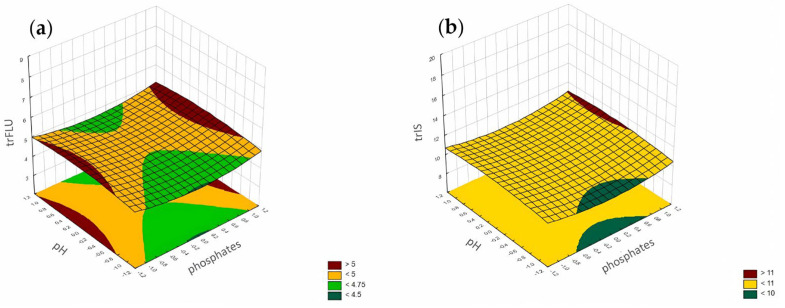
The RSM diagram for analysis of the retention time when analyzing pH level and phosphates for (**a**) FLU and (**b**) the IS.

The most significant factor influencing the retention time of FLU and the IS was the concentration of ACN (Figure 6). The increase in ACN's concentration led to a decrease in the retention times of both analytes. Analysis of the RSM diagrams for both analytes also showed that the retention of the analytes depended mainly on ACN. The pH level and phosphate buffer concentration also had a significant impact, but in comparison with ACN's influence, it was not so considerable (Figure 7, Figure 8 and Figure 9). The F value in the ANOVA also took the higher value for ACN for both analytes (Table 2).

The global ANN sensitivity analysis conducted for the retention times of FLU and the IS also indicated the ACN concentration as the most significant factor. For the IS, the concentration of phosphates and pH level were in accordance with the results of the DoE approach. For FLU, the remaining two factors were in the following order of significance: pH level and concentration of phosphates. For DoE, the order was different, being concentration of phosphates and then pH level.

#### 3.1.2. Tailing Factor

**Figure 10:**
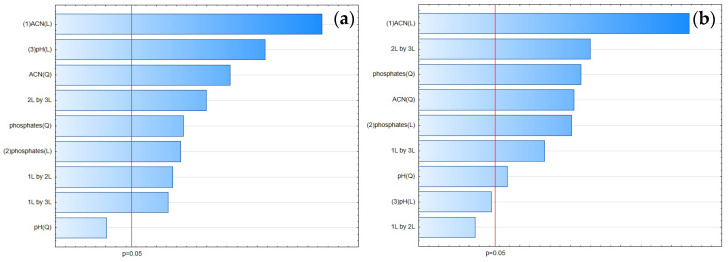
The Pareto chart for tailing factors of (**a**) FLU and (**b**) the IS.

**Figure 11:**
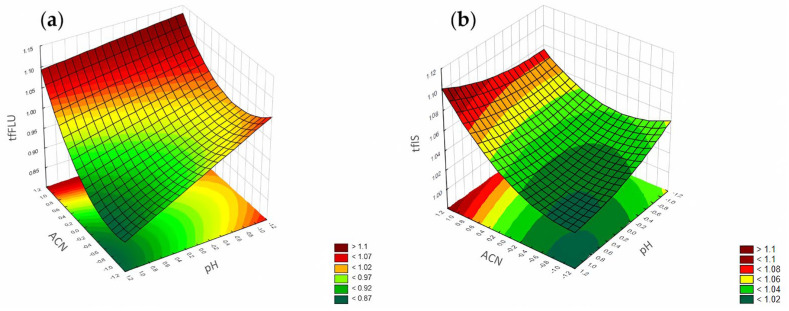
The RSM diagram for analysis of the tailing factors when analyzing ACN and the pH level for: (**a**) FLU and (**b**) the IS.

**Figure 12:**
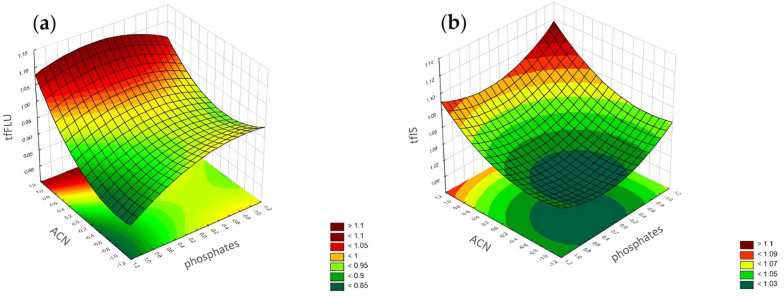
The RSM diagram for analysis of the tailing factors when analyzing ACN and phosphates for (**a**) FLU and (**b**) the IS.

**Figure 13:**
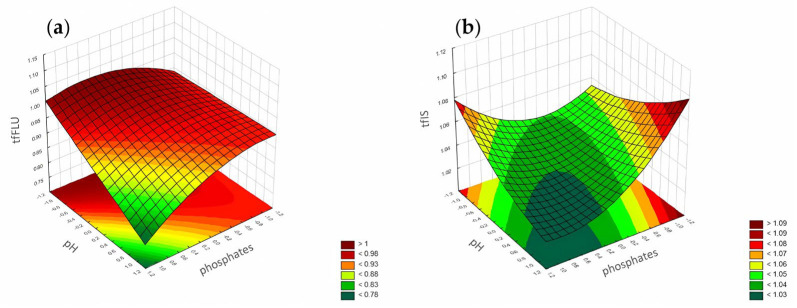
The RSM diagram for analysis of the tailing factors when analyzing the pH level and phosphates for (**a**) FLU and (**b**) the IS.

According to the Pareto chart (Figure 10), the most significant factor which influenced the tailing of the peaks was the concentration of ACN. However, in the case of FLU, the other factor was pH level, and the interaction of concentration of phosphates with pH level. For the IS, the interaction of pH level with concentration of phosphates also influenced the tailing of the peaks (Figure 11, Figure 12 and Figure 13).

Following the ANN approach, the ACN concentration was also most significant in the case of a tailing factor for both analytes. The remaining two variables for FLU were the pH level and concentration of phosphates. For the IS, it was the concentration of phosphates and pH level. 

#### 3.1.3. Peak Height

**Figure 14:**
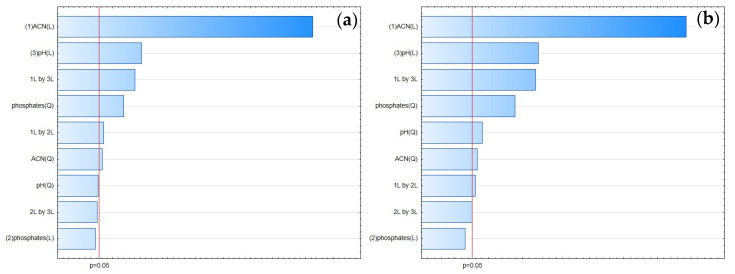
The Pareto chart for peak height for (**a**) FLU and (**b**) the IS.

**Figure 15:**
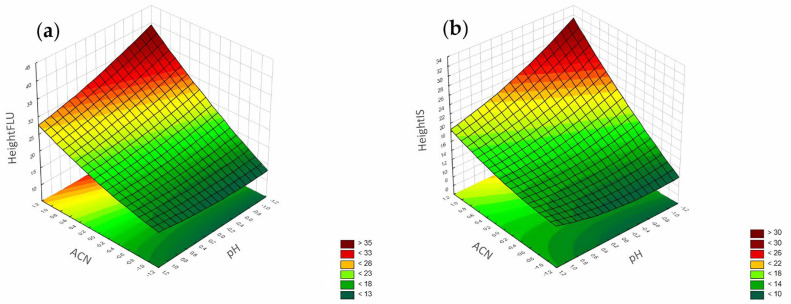
The RSM diagram for analysis of the peak height when analyzing ACN and pH level for (**a**) FLU and (**b**) the IS.

**Figure 16:**
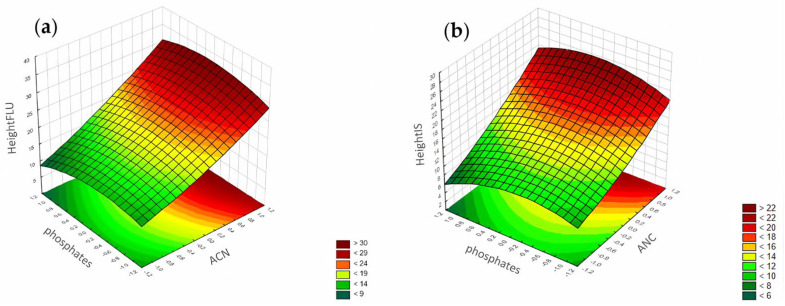
The RSM diagram for analysis of the peak height when analyzing ACN and phosphates for (**a**) FLU and (**b**) IS.

**Figure 17:**
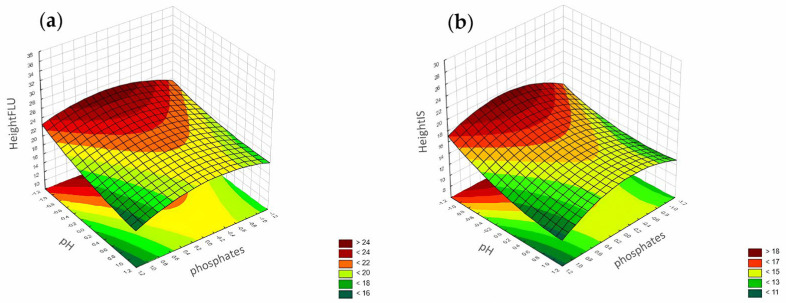
The RSM diagram for analysis of the peak height when analyzing pH level and phosphates for (**a**) FLU and (**b**) the IS.

According to the Pareto chart, the most significant factor influencing the peak height was the concentration of ACN (Figure 14). In turn, analysis of the RSM diagrams showed that both ACN and the pH level as well as their interaction were the most significant factors influencing the peak height for both analytes (Figure 15, Figure 16 and Figure 17).

For the peak height, the ANN analysis revealed that the most significant factor was acetonitrile. The remaining two were the pH level and phosphates for both analytes. In this case, the data were in accordance with the results obtained for the DoE analysis.

#### 3.1.4. Final Chromatographic Conditions

**Figure 18:**
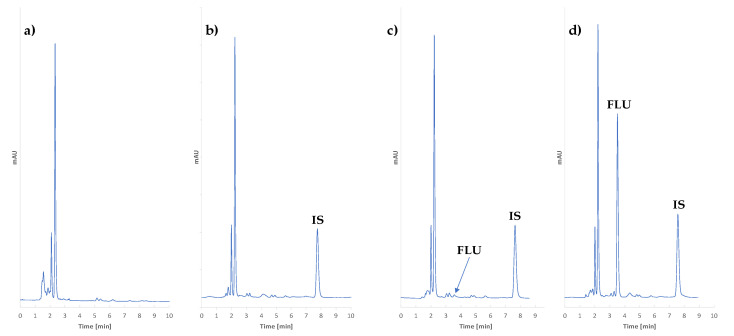
The chromatograms of the (**a**) blank sample, (**b**) zero sample, (**c**) 0.5 mg/L (LLOQ) FLU sample, and (**d**) 75.0 mg/L FLU sample.

Chromatographic separation was performed at room temperature. The mobile phase was prepared by mixing ACN and 20 mmol NaH_2_PO_4_ solution at a ratio of 23:77 and adjusting it to a 5.5 pH level with NaOH solutions. The flow rate and wavelength were set to 1 mL/min and 261 nm, respectively. The retention time for FLU was t_rFLU_ = 3.5 min., and for the IS, it was t_rIS_ = 7.6 min. The chromatograms are presented in (Figure 18). In the chromatograms, the total resolution of the analytes from the other signals was observed, and they did not interfere with the signals of endogenous substances.

### 3.2. LLE Optimization

**Figure 19:**
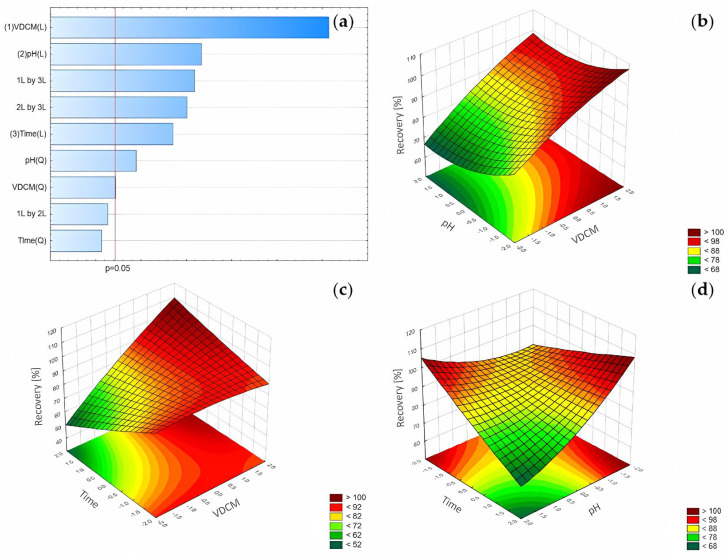
(**a**) The Pareto chart for recovery of FLU analysis and the RSM diagram for recovery of FLU when analyzed: (**b**) pH and VDCM, (**c**) VDCM and time, and (**d**) pH and time.

The analysis of the RSM diagrams showed that the recovery of FLU increased with the increase in VDCM, regardless of the applied pH level (Figure 19). The highest recovery was observed for VDCM of at least 3.5 mL and a pH range of 4.0–5.5. The combination of at least 3.5 mL VDCM and a shaking time of at least 15 min resulted in the highest recovery values, reaching 100%. The pH-time chart also confirms that the samples should be shaken for at least 15 min at a pH of 5.5. Based on these observations, the highest recovery was observed for 3.5 mL VDCM, a pH of 5.5, and a shaking time 15 min. The predicted theoretical value of the recovery was approximately 100%, and the observed value was 99%.

In the case of ANN recovery analysis, the most significant factor was the volume of the extracting agent. The subsequent factors ranked in order of decreasing importance were the pH level and shaking time. The following results are consistent with the results obtained with the regression analysis.

A summary of the comparison of all results from the DoE and ANN analyses is presented in Table 8.

### 3.3. Method Validation and Its Application in PK Analysis

The method was validated toward the CV and RE (inter- and intraday), linearity, and stability. The regression was linear within the following concentration range: 0.5–100.0 mg/L. This was confirmed with the correlation coefficient value of 0.997. The statistical analysis confirmed that the intercept was not statistically significant.

The intra- and interday RE ranged from 0.08 to 4.04% and from 0.06 to 10.33%, respectively. In the case of the CVs, it was from 1.97 to 6.14% and from 0.68 to 6.56% for the intra- and interday periods, respectively (Table 6). These values did not exceed 15%, and for LLOQ, it was 20%. This implies that the method was accurate and precise.

Carryover was not observed for either FLU or the IS.

The recovery value was approximately 100%, which indicates that the analyte was entirely extracted from the matrix. It was not concentration-dependent; a similar value was observed for low and high concentrations. For comparison, the recovery with DCM liquid-liquid extraction in the assay performed by Kim et al. reached ~83%, but it was tested only in a wide range of FLU concentrations (0.15, 4.0, and 8.0 mg/L) [17]. In turn, Gordien et al. reported ~90% recovery based on solid-phase extraction [18].

The stability tests confirmed the stability of FLU. The analyte remained stable at room temperature for up to 24 h as well as in low temperatures (Table 7). The results of the freeze-thaw analysis confirmed that the thawing of the sample did not have an impact on the stability of FLU. The results from the stability tests obey the EMA recommendations. The RE did not exceed ±15%. The stability tests conducted after one year of storage for the plasma sample with fluconazole revealed that for storage at −25 °C and −80 °C, the RE was higher than 15%. In the cases of storage at −25 °C and −80 °C, the value did not exceed 22% or 19%, respectively. This implies that the samples should be analyzed at a temperature of −25 °C or lower within one month.

The preliminary PK examination confirmed the practical application of the method in pharmacokinetic studies (see Section 2.4). The investigated range allowed for method implementation in the pediatric population, where FLU’s maximum concentration (C_max_) depends on age and fluctuates between 2.9 and 14.1 mg/L [4]. On the other hand, the high upper limit of quantification (ULOQ = 100 mg/L) allows our method to be used even among patients treated in intensive care units, for whom FLUs may reach up to approximately 80 mg/L [19].

The undoubtful advantage of our method is the relatively small required volume for the plasma sample (200 µL) to perform determination of the FLU concentration. In comparison, in the method applied by Kim et al., it is 500 µL, and in the study by Liew et al., it was as much as 1 mL [17,20]. The volume of the plasma sample being as small as possible was one of the main goals during the development process of our method. This was caused by the restrictions of bioethical committees, which are strict regarding the sample volumes taken from pediatric patients and aim to minimize their volume. Regarding the speed of analysis, our method is slightly faster than other developed methods, allowing us to save time and reagents [17,20,21]. Another advantage is the wide linear concentration range from 0.5 to 100 mg/L, which is one of the widest described in the literature and may be used in the FLU concentration’s determination in the adult intensive care unit population. As a comparison, in the method applied by Kim et al., it was from 0.05 to 10 mg/L, and for Liew et al., it was from 0.125 to 10 mg/L [17,20]. In the case of the Singh et al. approach, the range was wider (0.1–40 mg/L), but the authors developed a method using blood in plasma simulant-phosphate buffer saline (PBS) [21]. It is worth underlining that the designed optimization process allowed for the recovery of both FLU and the IS at a rate of approximately 100%, while in the method of Kim et al., they were approximately 80% for FLU and only 50% for IS [17]. Similar recovery percentages proving the possibility of reaching almost 100% were observed in the studies by Liew et al. and Singh et at. [20,21]. With reference to the choice of IS, phenacetin is a reasonably good choice because it is no longer used in clinical practice, relatively cheap, and readily available. Moreover, according to the Kim et al. reports, it was one of the first and most widely chosen ISs for FLU determination method development [17].

The next step after development of the method was determination of the FLU concentrations in the collected pediatric patients.

## 4. Materials and Methods

### 4.1. Reagents and Materials

Fluconazole (MedChemExpress; Monmouth Junction, NJ, USA) and phenacetin as the internal standard (IS) (HPLC grade, Sigma-Aldrich; Steinheim, Germany) were used in this analysis. Acetonitrile (ACN) and methanol (MeOH) of liquid chromatography-mass spectrometry grade were purchased from Merck (Darmstadt, Germany). Dichloromethane (DCM) and phosphates (NaH_2_PO_4_) were obtained from Sigma-Aldrich (Steiheim, Germany). Sodium hydroxide (NaOH) and boric, phosphoric, and acetic acid were purchased from Avantor Performance Materials (Gliwice, Poland). A Millipore system (Direct Q3, Millipore, Burlington, MA, USA) was utilized to deionize, distill, and filter the water used during analysis. The Regional Blood Donation Center in Poznan, Poland provided drug-free human plasma. The HPLC analysis was performed using an Xterra RP-18 column (160 × 4.6 mm, 3.5 µm, Waters, Milford, MA, USA) with an Xterra RP-18 pre-column (3.9 × 5 mm, 3.5 µm, Waters, USA) using an Agilent Technologies 1220 Infinity LC chromatograph (Santa Clara, CA, USA).

### 4.2. Solutions

The FLU stock solutions (10 mg/mL) and IS (1 mg/mL) were prepared by dissolving in MeOH a proper amount of solid substance in a 10 mL volumetric flask. The FLU standard solutions were prepared using serial dilutions from stock solutions with MeOH. The standard solution of the IS (20 mg/L) was achieved similarly. The used concentrate of Britton–Robinson buffer (BRB), a universal buffer for pH = 2–12, was prepared by mixing the proper amount of boric acid, phosphoric, and acetic acid in a volumetric flask to reach the final concentrations of the analytes (0.4 M). We used the concentrated buffer (10 times) to overcome the buffer capacity of the plasma. The desired pH value was obtained by adding NaOH.

### 4.3. Optimization Procedures

The data were analyzed with Statistica 13.3 (TIBCO Software Inc.; Palo Alto, CA, USA). According to the design of experiments (DoE) approach, the analyzed dependent variables were described quantitively with the second-order polynomial equation. The regression coefficients (R^2^ and R^2^_adj_) were calculated using analysis of variance (ANOVA). Additionally, the randomization procedure was applied. Each experiment was repeated twice. The levels of the independent variables were coded according to the work of Bezerra et al. [22]. In addition to the classic optimization procedure, we preliminarily performed the testing with an artificial neural network (ANN). The applied artificial neural networks (ANNs) consisted of the input, an output layer, and one hidden layer. For each investigation, there were 50 different ANNs tested with a maximum of 10 hidden layer neurons. The maximal number of training epochs was set to 300. An R^2^ of error of ~1 was considered the stop criterion. The ANN was trained with the feedforward and backpropagation processes and a supervised learning algorithm until the mean square error took on the lowest value. A multilayer perceptron (MLP) was applied for the construction of ANNs. The training of the MLP was conducted in the following steps: provision of the data to the input layer, obtaining the result in the output layer and evaluating its error, backpropagation to the hidden layer, and the process starting again until the error took on the lowest value.

#### 4.3.1. Mobile Phase Optimization

The mobile phase composition was optimized with the Box–Behnken design, which is a suitable model for analysis [15]. The following independent variables were optimized: content of ACN, pH level, and concentration of NaH_2_PO_4_. The BBD matrix is presented in Table 9, and the coded levels are presented in Table 10. The analyzed responses (dependent variables) were the retention time, peak height, and tailing factor for both FLU and the IS.

For the optimization of the mobile phase using an ANN, the input layer contained three neurons for the concentration of acetonitrile, pH level, and concentration of phosphates. The output layer contained one neuron, which was the retention time of the analyte, peak height, or tailing factor.

#### 4.3.2. Liquid-Liquid Extraction Optimization

The recovery was optimized for liquid-liquid extraction (LLE) in DCM. The circumscribed central composite design (CCD) was applied in the optimization process as a suitable model [23,24] with the previously described ANN. The analyzed independent variables were the volume of DCM (VDCM), pH level, and shaking time. The star points were denoted as |α| = 1.67, and the remaining levels were −1, 0, and 1. For the ANN approach, the input layer was three neurons representing tested independent variables. The analyzed response was the recovery of fluconazole (also one neuron as an output layer for the ANN approach). The design matrix is presented in Table 11. Each experiment was repeated twice, ensuring the reliability and robustness of the results. The levels of the independent variables are presented in Table 12. The recovery was tested for low and high concentrations (1.5 and 75.0 mg/L) for the optimized conditions to check whether it depended on the concentration.

The LLE was optimized on human plasma [23,24]. The sample was prepared according to the following procedure. First, 200 µL of plasma was added to 20 µL of FLU solution (100 mg/L), 20 µL of the IS (50 mg/L), and 500 µL of buffer, with a proper volume of DCM (according to Table 11 and Table 12). Then, the sample was shaken. The added VDCM, pH of the buffer, and time of shaking were defined in Table 12. After shaking, the samples underwent centrifugation to provide phase separation (5 min, 1300× *g*). The DCM layer was transferred to the tube and evaporated in the sample concentrator. The solid residue was dissolved in 50 µL of the mobile phase and transferred to vials, and 10 µL was injected into the HPLC column. Two sets of samples were prepared to evaluate the recovery. In the first one, FLU was extracted using the procedure described above. In the second one, it was added prior to injection onto the chromatographic system. FLU was dissolved in the mobile phase, and its concentration was the same as the theoretical concentration in the analyzed sample when the solid residue was dissolved in the mobile phase. In the case of the second set of samples, to maintain the constant volume, 20 µL of MeOH was added instead of the solution of the analyte.

### 4.4. HPLC Method Validation

The method was validated according to EMA criteria for specificity, selectivity, method linearity, range, accuracy, precision, carryover, and stability [25]. The concentration range for the calibration curve was 0.5–100.0 mg/L for FLU. The mean calibration curve was calculated based on five calibration curves. The measurements were performed within a day (intraday) and for five consecutive days (interday).

The inter- and intraday precision and accuracy measurements were performed for low (0.5 and 1.5 mg/L) and high (40.0 and 75.0 mg/L) concentrations. The analysis was performed based on five repetitions per condition. The precision was calculated as the coefficient of variation (CV) according to the following formula:

$$CV=\frac{SD}{\bar{X}}\times 100\%$$

where *SD* is the standard deviation and $\bar{X}$ is the mean value.

The accuracy was calculated as the relative error (RE) with the following formula:

$$RE=\left|\frac{c_{t}-c_{d}}{c_{t}}\right|\times 100\%$$

where *c_t_* is the theoretical concentration and *c_d_* is the determined concentration.

The samples were prepared under optimized conditions described in Section 3.2. and according to the procedure described in Section 4.3.2.

The stability in the plasma samples was determined for 24 h of storage in the autosampler, 3 h at the bench top and as a dry extract (room temperature), after three freeze-thaw cycles, and after 1 month and 1 year of storage at −25 and −80 °C (long-term stability). All of the mentioned stability procedures were performed for 1.5 and 75.0 mg/L of FLU, with three repeats for each concentration. Specificity was assessed for drugs which were often co-administered with FLU in a hemato-oncologic pediatric population, such as furosemide, meropenem, ceftriaxone, cefotaxime, paracetamol, ibuprofen, metamizole, desloratadine, sertraline, prednisone, gabapentin, and omeprazole. The final concentrations of the drugs mentioned above were related to their maximum plasma concentrations (C_max_).

### 4.5. Application to Pharmacokinetic Studies

The method was evaluated by analyzing 12 plasma samples collected from three hemato-oncologic pediatric patients (6–11 years) treated with intravenous fluconazole (200–300 mg per 24 h in 30 min intervals). Blood samples were collected in heparinized tubes and centrifuged at 3000× *g* for 15 min. Plasma samples were stored at −25 °C until analysis. The study protocol was approved by the Poznan University of Medical Sciences Bioethics Committee (agreement 820/21), and informed consent from the patients and patients’ parents was obtained. PK analysis was performed based on a one-compartment model using PKanalix 2024R1 (Lixoft SAS, a Simulations Plus company, Lancaster, CA, USA) software. The clearance (CL) and volume of distribution (Vd), as the investigated parameters, were averaged due to the small number of samples per patient.

## 5. Conclusions

The central composite design and Box–Behnken design in the design of experiments approach and artificial neural networks are useful statistical tools in analytical optimization processes. They are helpful in the optimization of several dependent variables necessary for efficient chromatographic analysis. As an addition to and support of statistical methods, artificial neural networks proved to be an equally valuable tool in optimization of the procedure of chromatographic separation by HPLC as well as sample preconditioning. It is crucial to highlight that both mentioned solutions allow for reducing the use of resources spent on process development and saving time in the investigation of analytical conditions. The proposed FLU determination method met the validation objectives, and it is characterized by flexibility in the therapeutic drug monitoring process in different populations due to a wide range of determined concentrations. The short time and analysis imply that this method might be used in drug-level analysis for clinical purposes.

## Figures and Tables

**Table 1:** The statistical analysis of the investigated models regarding the chromatographic factors.

Analysed Factor	FLU	IS
Retention time		
R^2^	0.9990	0.9985
R^2^_adj_	0.9973	0.9959
Peak height		
R^2^	0.9958	0.9947
R^2^_adj_	0.9881	0.9875
Tailing factor		
R^2^	0.9975	0.9837
R^2^_adj_	0.9930	0.9544

**Table 2:** The results of ANOVA analysis for the retention time of FLU and IS.

Variable	FLU		IS	
	F	*p*	F	*p*
ACN (L)	42,925.99	<0.0001	384,595.0	<0.0001
ACN (Q)	3196.67	0.0003	16,334.0	<0.0001
Phosphates (L)	3.10	NS	53.4	0.0182
Phosphates (Q)	213.56	0.0047	1645.5	0.0006
pH (L)	32.35	0.0295	417.1	0.0024
pH (Q)	56.94	0.0171	401.6	0.0025
ACN × phosphates	0.61	NS	2.1	NS
ACN × pH	62.44	0.0156	166.5	0.0060
pH × phosphates	1.30	NS	29.3	0.0325

L =linear; Q = quadratic term; NS- not significant.

**Table 3:** The results of ANOVA analysis for a tailing factor of FLU and the IS.

Variable	FLU		IS	
	F	*p*	F	*p*
ACN (L)	458.92	<0.0001	473.78	<0.0001
ACN (Q)	151.91	<0.0001	107.58	0.0001
Phosphates (L)	54.79	0.0007	102.35	0.0002
Phosphates (Q)	58.85	0.0006	121.85	0.0001
pH (L)	248.44	<0.0001	4.87	NS
pH (Q)	0.0097	NS	14.37	0.0128
ACN × phosphates	44.23	0.0012	0.37	NS
ACN × pH	37.92	0.0016	55.61	0.000684
pH × phosphates	99.51	0.0002	143.19	0.0001

L = linear; Q = quadratic term; NS – not significant.

**Table 4:** The results of ANOVA analysis for the peak height of FLU and the IS.

Variable	FLU		IS	
	F	*p*	F	*p*
ACN (L)	1703.31	<0.0001	1176.00	<0.0001
ACN (Q)	9.88	0.0256	10.94	0.0213
Phosphates (L)	3.29	NS	2.30	NS
Phosphates (Q)	49.79	0.0009	80.08	0.0003
pH (L)	104.56	0.0001	153.23	<0.0001
pH (Q)	5.47	NS	16.75	0.0094
ACN × phosphates	11.32	0.0200	9.24	0.0288
ACN × pH	81.76	0.0003	142.28	<0.0001
pH × phosphates	4.92	NS	6.61	0.0499

L = linear; Q = quadratic term, NS – not significant.

**Table 5:** Results of ANOVA for recovery.

Variable	F	*p*
VDCM (L)	2645.476	0.0004
VDCM (Q)	19.211	0.0483
pH (L)	544.478	0.0018
pH (Q)	79.420	0.0124
Time (L)	291.608	0.0034
Time (Q)	1.572	NS
VDCM × pH	6.862	NS
VDCM × time	472.952	0.0021
pH × time	404.726	0.0025

L = linear; Q = quadratic term, NS – not significant.

**Table 6:** The precision and accuracy results for inter- and intraday assays for FLU.

Concentration (mg/L)	Intraday		Interday	
	CV (%]	RE (%)	CV (%)	RE (%)
75.0	2.46	0.09	2.41	0.06
40.0	1.97	0.32	2.20	0.22
1.5	6.14	4.04	6.56	0.75
0.5	5.86	0.08	0.68	10.33

**Table 7:** The results of the stability assay of FLU in plasma samples.

Condition	Concentration (mg/L)	Mean Determined Concentration (mg/L)	RE (%)
Autosampler (room temperature for 24 h)	1.5	1.69	12.34
	75.0	78.03	4.04
Bench top (room temperature for 3 h)	1.5	1.72	14.92
	75.0	72.07	3.91
Freeze-thaw	1.5	1.62	8.20
	75.0	74.75	0.33
Dry extract (room temperature for 3 h)	1.5	1.66	10.38
	75.0	73.68	1.76
1 month (−25 °C)	1.5	1.67	11.31
	75.0	78.23	4.31
1 month (−80 °C)	1.5	1.72	14.43
	75.0	19.56	6.08
1 year (−25 °C)	1.5	1.25	16.65
	75.0	58.77	21.64
1 year (−80 °C)	1.5	1.28	14.58
	75.0	60.09	19.00

**Table 8:** Direct comparison of the results of the DoE and ANN analyses.

Parameter	Significance Order	DoE Approach		ANN Approach	
		FLU	IS	FLU	IS
Retention time	Most	ACN	ACN	ACN	ACN
	Medium	NaH_2_PO_4_	NaH_2_PO_4_	pH	NaH_2_PO_4_
	Least	pH	pH	NaH_2_PO_4_	pH
Tailing factor	Most	ACN	ACN	ACN	ACN
	Medium	pH	NaH_2_PO_4_	pH	NaH_2_PO_4_
	Least	NaH_2_PO_4_	pH	NaH_2_PO_4_	pH
Peak height	Most	ACN	ACN	ACN	ACN
	Medium	pH	pH	pH	pH
	Least	NaH_2_PO_4_	NaH_2_PO_4_	NaH_2_PO_4_	NaH_2_PO_4_
Recovery	Most	VDCM	–	VDCM	–
	Medium	pH	–	pH	–
	Least	Time	–	Time	–

**Table 9:** BBD matrix with investigated factors for optimization of the mobile phase.

ACN	NaH_2_PO_4_	pH
1	0	−1
0	−1	−1
−1	−1	0
0	0	0
0	0	0
1	−1	0
−1	0	1
0	−1	1
0	1	−1
−1	0	−1
−1	1	0
0	0	0
1	0	1
1	1	0
0	1	1

**Table 10:** The coded levels for ACN, phosphate concentration, and pH level.

Independent Variable	Levels		
	−1	0	1
ACN (%)	15	20	25
NaH_2_PO_4_ (mmol)	5	15	25
pH	4.0	5.5	7.0

**Table 11:** The circumscribed CCD matrix with investigated factors in the recovery optimization.

VDCM	pH	Shaking Time
1	1	1
1	−1	1
0	0	1.67
1.67	0	0
0	1.67	0
−1.67	0	0
1	1	−1
0	−1.67	0
1	−1	−1
−1	−1	1
−1	1	−1
−1	1	1
0	0	0
0	0	−1.67
−1	−1	−1

**Table 12:** The coded levels for VDCM, pH level, and shaking time.

Independent Variable	Levels				
	−1.67	−1	0	1	1.67
VDCM (mL)	0.8	1.46	2.50	3.54	4.20
pH	4.00	5.55	8.00	10.45	12.00
Shaking time (min)	1.60	4.84	10.00	15.15	18.40

## Data Availability

The data presented in this study are available from the corresponding author upon reasonable request.

## Supplementary Materials

The following supporting information can be downloaded at https://www.mdpi.com/article/10.3390/ph17121679/s1. Table S1: The results of measurements for chromatographic separation optimization analysis. Table S2: The results of measurements for recovery optimization analysis.
