# Supplementary material for: The Application of the Design of Experiments and Artificial Neural Networks in the Development of a Fast and Straightforward HPLC-UV Method for Fluconazole Determination in Hemato-Oncologic Pediatric Patients and Its Adaptation to Therapeutic Drug Monitoring
Source: Pharmaceuticals (Basel). 2024 Dec 12;17(12):1679. doi: 10.3390/ph17121679 (PMC11679493; doi:10.3390/ph17121679)
Supplement: Supplementary file 1 [file pharmaceuticals-17-01679-s001.zip › pharmaceuticals-3339278-supplementary.pdf]

Supplementary Materials for "The application of the design of experiment and artificial neural networks in the development of a fast and straightforward HPLC-UV method for fluconazole determination in haemato-oncologic pediatric patients and its adaptation to therapeutic drug monitoring"

Table S1. The results of measurements for chromatographic separation optimization analysis.

Coded levels			FLU retention time	IS retention time	FLU peak height	IS peak height	FLU tailing factor	IS tailing factor
ACN	Phosphates	pH						
1.00000	0.00000	-1.00000	3.230	6.675	35.710	27.920	1.090	1.067
0.00000	-1.00000	-1.00000	4.830	10.420	21.700	16.300	0.960	1.046
-1.00000	-1.00000	0.00000	8.705	17.570	14.270	10.200	0.970	1.050
0.00000	0.00000	0.00000	4.800	10.300	21.610	15.750	0.960	1.031
0.00000	0.00000	0.00000	4.730	10.340	21.610	15.740	0.970	1.031
1.00000	-1.00000	0.00000	3.370	7.025	28.110	19.380	1.042	1.098
-1.00000	0.00000	1.00000	8.365	16.840	15.560	12.590	0.890	1.016
0.00000	-1.00000	1.00000	4.920	10.540	18.710	13.090	0.940	1.080
0.00000	1.00000	-1.00000	4.825	10.455	22.970	17.270	1.002	1.061
-1.00000	0.00000	-1.00000	7.935	16.120	14.330	10.760	1.020	1.046
-1.00000	1.00000	0.00000	8.730	17.615	10.960	8.030	0.870	1.030
0.00000	0.00000	0.00000	4.760	10.300	21.520	15.760	0.960	1.031
1.00000	0.00000	1.00000	3.105	6.799	26.995	18.800	1.060	1.080
1.00000	1.00000	0.00000	3.450	7.138	28.500	20.000	1.050	1.075
0.00000	1.00000	1.00000	4.995	10.825	17.540	11.700	0.820	1.026

ACN – acetonitrile, FLU – fluconazole, IS – internal standard

Table S2. The results of measurements for recovery optimization analysis.

Coded levels			Recovery [%]
VDCM	pH	Time	
1	1	1	89.22
1	-1	1	102.48
0	0	1.67	82.85
1.67	0	0	96.15
0	1.67	0	84.31
-1.67	0	0	72.17
1	1	-1	94.21
0	0	0	86.09
0	-1.67	0	95.32
1	-1	-1	91.66
-1	-1	1	80.94
-1	1	-1	86.37
-1	1	1	67.21
0	0	0	86.04
0	0	0	86.94
0	0	-1.67	90.26
-1	-1	-1	87.10

VDCM – volume of dichloromethane
